# Bioengineered heart valves for personalized therapy: advances in manufacturing and clinical challenges

**DOI:** 10.3389/fbioe.2025.1714534

**Published:** 2026-01-12

**Authors:** Mahnoor Saeed, Kamran A. Khan

**Affiliations:** 1 Department of Aerospace Engineering, Khalifa University of Science and Technology, Abu Dhabi, United Arab Emirates; 2 Advanced Digital and Additive Manufacturing Group, Khalifa University of Science and Technology, Abu Dhabi, United Arab Emirates

**Keywords:** precision medicine, immunomodulation, cardiovascular bioengineering, advanced manufacturing, scaffold engineering

## Abstract

Valvular heart diseases (VHDs) remain a significant clinical challenge, with mechanical and bioprosthetic valves offering only temporary solutions and failing to address long-term complications such as structural degradation, immune rejection, and the inability to dynamically remodel. Over the past two decades, tissue-engineered heart valves (TEHVs) have emerged as a promising alternative, combining biomaterials and patient-specific strategies to overcome these limitations. However, persistent issues, such as immune rejection, poor hemocompatibility, and inconsistent remodeling - continue to hinder clinical translation. Recent advances in immunomodulation, scaffold engineering, and personalized therapies show promise in mitigating these challenges, yet a fully integrated, comprehensive strategy remains elusive. This review critically explores the convergence of TEHVs and immunomodulation, focusing on how biomaterial-based immune engineering, nanoparticle-driven tolerance strategies, and advanced scaffold design can reshape heart valve therapy. By synthesizing recent innovations and highlighting key translational gaps, this paper lays the groundwork for a new generation of TEHVs that integrate, adapt, and regenerate, moving beyond passive mechanical replacements toward truly personalized cardiovascular solutions.

## 1 Introduction

Valvular dysfunction remains a persistent challenge in cardiovascular medicine, contributing significantly to the global burden of heart disease. Cardiovascular diseases (CVDs) account for an estimated 17.9 million deaths annually (WHO), with valvular heart diseases (VHDs) forming a critical subset arising from conditions such as atherosclerosis, rheumatic heart disease, and endocarditis (Di Francesco et al., 2023; Copes et al., 2019; Kitsuka et al., 2022; Tornos-Mas, 1994). These conditions cause structural damage to heart valves, impairing function and, if left untreated, significantly increasing mortality due to heart failure and arrhythmias (Tornos-Mas, 1994). Current interventions - mechanical and bioprosthetic valves - offer temporary relief but are limited in durability and adaptability. This review argues that bioengineered heart valves - fabricated using advanced manufacturing techniques and integrating immunomodulatory strategies with patient-specific approaches - represent the next Frontier in cardiovascular therapy.

Mechanical and bioprosthetic valves have been the standard of care in valve replacement therapy for decades. While mechanical valves offer long-term durability, they require lifelong anticoagulation therapy, increasing the risk of hemorrhagic complications (Frankel and Nguyen, 2021). Bioprosthetic valves avoid this requirement but are susceptible to gradual structural deterioration, often necessitating reintervention within 10–15 years (David, 2021). Most importantly, neither option can adapt to the host environment or promote tissue regeneration, leading to long-term complications and repeat surgeries.

Tissue-engineered valves have emerged as a promising alternative, yet they still face critical challenges - most notably, immune rejection and the inability to remodel dynamically. Many current approaches rely on decellularized matrices or synthetic scaffolds seeded with stem cells; however, without active immunomodulation, these constructs can trigger chronic inflammation or fibrosis, compromising their long-term viability (Liu et al., 2020). This review explores how integrating immunomodulatory strategies into bioengineered heart valves can help overcome these limitations, offering a patient-specific and durable solution.

Although patient-specific approaches, such as the use of autologous induced pluripotent stem cells (iPSCs) and mesenchymal stem cells (MSCs), have shown promise, they do not fully eliminate immune-related complications (Ashorobi et al., 2024; Misfeld and Sievers, 2007). Even autologous cells can be rejected if the surrounding microenvironment does not support immune tolerance. A transformative strategy for bioengineered heart valves involves the development of scaffolds that actively modulate immune responses, thereby promoting long-term graft acceptance.

**FIGURE 1:**
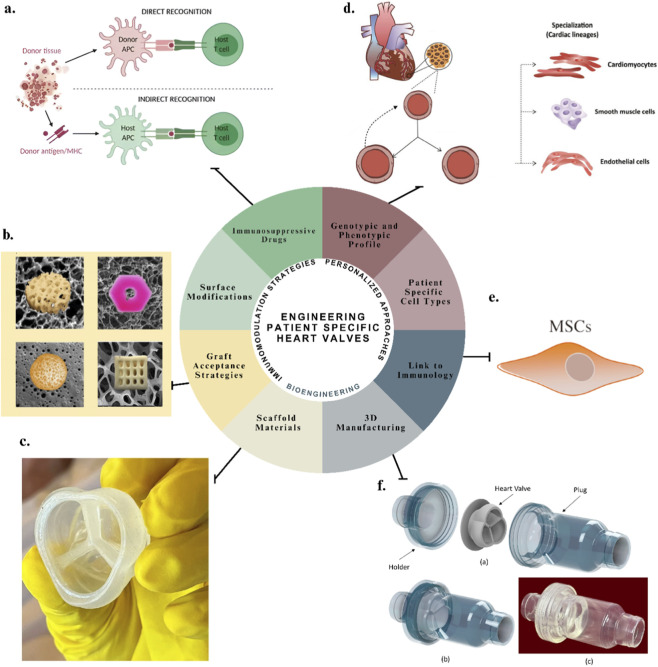
Engineering patient-specific heart valves: an integrated approach combining scaffold design, personalized cell sources, and immunomodulatory strategies to promote graft integration, regeneration, and long-term function. **(a)** Schematic representation of personalized cell sourcing using autologous stem cells (Claeys et al., 2019); **(b)** workflow of patient-specific tissue engineering integrating imaging, modeling, and bioprinting (Jahangirian et al., 2019); **(c)** immunomodulatory nanoparticle strategies for promoting graft tolerance (Angelo et al., 2025); **(d)** decellularized scaffold preserving extracellular matrix architecture for valve regeneration (Leite et al., 2015); **(e)** biomechanical bioreactor system used for conditioning tissue-engineered valves (Nikolskii et al., 2007); and **(f)** integration of immunomodulatory cues into engineered heart valve constructs (Angelo et al., 2025).

Emerging research in biomaterial-based immunoengineering, made evident in Figures 1a,f, along with nanoparticle vaccines highlights the potential for intentional immunomodulation in tissue engineering (Goodwin and Biechler, 2019). Strategies such as tolerogenic nanoparticles delivering targeted immunosuppressants, or MHC-matched allogeneic cell therapies, offer promising avenues for developing heart valves that integrate seamlessly into the host system rather than being treated as foreign objects (Spicer et al., 2014). This review critically examines these innovations and explores their potential role in the development of next-generation, patient-specific heart valves.

Existing studies have reviewed localized tissue-engineered constructs as defect-specific solutions (Jeng et al., 2013). In contrast, heart valves exist in a uniquely challenging environment, with a need for maintaining hemocompatibility, high tensile and compressive stresses (Butcher et al. 2008). The scientific insight of this work is that successful next-generation tissue-engineered heart valves (TEHVs) cannot be achieved by scaffold design or cell sourcing alone. There are three areas that must be coupled to achieve the next-generation of tissue engineered heart valves: patient specific cell sourcing, immunomodulation strategies, and finally mechanically and hemodynamically competent scaffolds in adherence to ISO-5840 standards.

Despite progress within in each of these respective fields, the coupling of these three fields is crucial at this point to be able to address many of the issues associated with current TEHVs.

As illustrated in Figure 1, this review aims to highlight a future where heart valve replacements, achieved through advanced manufacturing techniques and the integration of bioengineering with immunomodulation, not only adapt and integrate but also regenerate - offering a durable cardiovascular solution that goes beyond passive mechanical substitutes.

**FIGURE 2:**
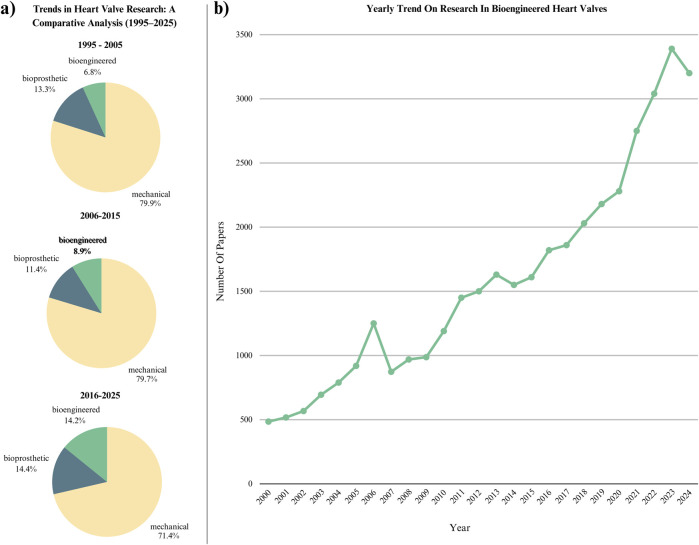
Comparative trends in heart valve research from 1995 to 2025 based on Google Scholar publication counts. **(a)** Proportional distribution of publications on mechanical, bioprosthetic, and bioengineered heart valves, showing a gradual shift towards tissue-engineered approaches; **(b)** yearly publication growth of bioengineered valve research, highlighting a sharp increase in interest over the past decade, particularly after 2020. Based on Google Scholar publication counts. Data reflect proportions of mechanical, bioprosthetic, and bioengineered valve studies (left), and yearly bioengineered valve publication growth (right). Search conducted April 2025 with citations included and patents excluded.

Figure 2 highlights the shift from traditional transplantation or mechanical-based heart valve replacement strategies to tissue engineering. With research in this field surging in 2023 and increasing approximately sevenfold over the past 25 years, tissue engineering has become a critical area of focus and development. However, the future of these advancements will depend on the strategies employed to support tissue-engineered constructs, particularly in the areas of immunomodulation and precision medicine.

## 2 Fundamentals of heart valve biology

### 2.1 Anatomy and function of heart valves

The heart contains four valves: two atrioventricular valves and two semilunar valves. The atrioventricular valves - commonly known as the tricuspid and mitral (or bicuspid) valves - are positioned between the right ventricle and right atrium, and the left ventricle and left atrium, respectively. Chordae tendineae and papillary muscles help maintain valve closure during ventricular contraction. The semilunar valves, consisting of the pulmonary and aortic valves, are located between the right ventricle and pulmonary artery, and the left ventricle and aorta, respectively. The semilunar valves have three crescent-shaped cusps, while the atrioventricular valves have two or three leaflets (Spicer et al., 2014). The complexity of these structures is remarkable, and molecular analysis of their components can enhance treatment strategies.

Heart valves are composed of three tissue layers: the ventricularis, spongiosa, and fibrosa. The ventricularis, the outermost layer, provides structural support and elasticity. The spongiosa, the middle layer, contains proteoglycans, glycosaminoglycans, and loosely arranged collagen fibers, which help facilitate shock absorption. The fibrosa, the innermost layer, anchors the valve to surrounding structures by extending into the cardiac skeleton (Ashorobi et al., 2024; Misfeld and Sievers, 2007).

The annulus, a ring of dense fibrous tissue rich in collagen, supports the base of each valve and maintains the shape of the valve orifice. Chordae tendineae, composed of collagen and elastin fibers, connect the atrioventricular valve leaflets to the papillary muscles, which are made of cardiac muscle tissue and attached to the ventricular walls. During ventricular contraction, the papillary muscles contract, tensing the chordae tendineae to stabilize the valve leaflets. Both sides of the valve leaflets are lined with endothelial cells, providing a smooth surface that minimizes friction and prevents blood clot formation.

As discussed in “Clinical Anatomy and Embryology of Heart Valves” (pp. 1–12), “*Malformation and dysfunction of these complex structures result in potentially fatal pathologies.”* (Goodwin and Biechler, 2019). This emphasizes the importance of developing long-term, comprehensive solutions to valvular dysfunction.

### 2.2 Common heart valve diseases and their impact

#### 2.2.1 Types of heart valve diseases

Previous research has predominantly focused on four major types of VHDs: aortic stenosis, mitral regurgitation, mitral stenosis, and aortic regurgitation (Maganti et al., 2010).

Aortic Stenosis (AS) is characterized by the narrowing of the aortic valve opening, which restricts blood flow from the heart to the rest of the body. This VHD is described as “*not yet [having] an effective preventive approach*” (Guedeney and Collet, 2022), and severe cases are estimated to affect up to 0.5% of the U.S. population - approximately 1.66 million people - highlighting its significant prevalence (Ancona and Comenale Pinto, 2020). Congenital AS is often caused by a bicuspid aortic valve, in which the valve has only two leaflets instead of the normal three (as discussed in Section 1.1) (Tsampasian et al., 2024). This structural anomaly can result in turbulent blood flow and early calcification (Courtney et al., 2023). Common symptoms include chest pain (angina), fainting (syncope), shortness of breath (dyspnea), and heart palpitations. As the condition progresses, it may lead to heart failure symptoms such as swelling in the ankles and legs (edema) and reduced physical activity tolerance (Steeds and Myerson, 2020).

Mitral Stenosis (MS) is similar in definition to AS, as both involve narrowing of a heart valve that restricts blood flow. However, MS differs from AS in several important ways. There are two primary types of MS. The first - and most globally prevalent - is Rheumatic Mitral Stenosis (RMS), typically resulting from rheumatic fever, which is often caused by *Streptococcus* bacterial infections. Rheumatic fever leads to structural abnormalities in the mitral valve, causing resistance to blood flow (Giannini et al., 2022). The *Streptococcus* bacteria trigger carditis, an inflammation of all three layers of the valve and its leaflets, which can also lead to calcification - the root of the second type of MS (Wunderlich et al., 2019). The second form is Degenerative Mitral Stenosis (DMS), often associated with Mitral Annulus Calcification (MAC) (Giannini et al., 2022; Abramowitz et al., 2015). While MAC more frequently leads to Mitral Regurgitation (MR) than MS, it is becoming a more notable cause of MS, particularly in aging populations, which are more common in developed countries. Common symptoms of mitral stenosis include shortness of breath (dyspnea), fatigue, and palpitations. As the disease advances, individuals may experience more severe symptoms such as swelling in the legs and ankles (edema) and reduced physical activity tolerance - symptoms that overlap with those seen in AS (Brown et al., 2023) (Amaral et al., 2019).

**FIGURE 3:**
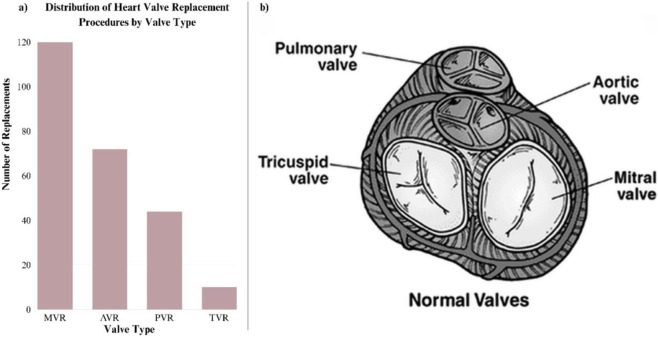
Heart valve disease overview and anatomical context. **(a)** Distribution of valve replacement procedures, illustrating the prevalence of aortic and mitral valve interventions (Samiei et al., 2014); **(b)** anatomical structure of the heart valves, highlighting the location and configuration of the aortic, mitral, pulmonary, and tricuspid valves (Singh et al., 2013). These visuals contextualize the clinical and anatomical basis of valvular heart diseases discussed in the section.

Aortic Regurgitation (AR) and Mitral Regurgitation (MR) are both forms of valvular heart disease characterized by the backflow of blood due to improper valve closure. MR and AR require the most valve replacements, which makes the large prevalence and degenerative effects of both VHDs clear (Figure 3a). Primary MR can result from several causes, including rheumatic fever (as discussed earlier), degenerative changes such as age-related deterioration and MAC, and Mitral Valve Prolapse, in which the mitral valve leaflets bulge backward into the left atrium during ventricular contraction (Pozzoli et al., 2016). In contrast, primary AR may be caused by chest trauma, congenital or acquired valvular disorders, and inflammatory conditions. One example is Primary Antiphospholipid Syndrome (PAPS), where antibodies bind to endothelial cells, promoting fibrosis and calcification. Other inflammatory causes include infective endocarditis and rheumatic fever, which also contribute to MR (Sakanoue et al., 1998). Both AR and MR commonly present with symptoms such as dyspnea, fatigue, palpitations, and angina. In severe cases, AR can progress to heart failure, while MR often leads to peripheral edema, particularly swelling in the legs and ankles (Marigliano et al., 2024; Cameron and Istrail, 2022).

The study *“Trends and Disparities in Valvular Heart Disease Mortality in the United States From 1999 to 2020*” highlights the distribution of deaths attributed to various subtypes of Valvular Heart Disease (VHD). Aortic Stenosis (AS) emerges as the most prevalent cause, accounting for 78% of all VHD-related deaths, followed by Mitral Regurgitation (MR) at 15%. Aortic Regurgitation (AR) and Mitral Stenosis (MS) contribute 4% and 3%, respectively. These findings underscore the substantial impact of AS on VHD mortality and reinforce the importance of early detection and targeted interventions to manage and mitigate its effects.

#### 2.2.2 Significance of VHDs and their effect on CVDs

Besides VHDs being extremely prevalent in countries such as the US, with over eight million (or 2.5% of the population) having a VHD (CDC) and 28.2% having a VHD in the UK, (Abramowitz et al., 2015), VHDs become increasingly problematic as they can progress to fatal CVDs.

One of the most common CVDs associated with VHDs is Atrial Fibrillation (AF). The structural and functional abnormalities of the heart valves, particularly the mitral and aortic valves, can create conditions that predispose patients to AF (Coffey et al., 2014) (Chan, 2024). VHDs may also cause heart failure, or strokes, as burden on the heart increases as it attempts to compensate for the dysfunctional valves through mechanisms such as hypertrophy and dilation. Over time, these compensatory responses lead to a decline in cardiac function and eventually heart failure (Nkomo et al., 2006). However, VHDs can predispose patients to many other CVDs, depending on their adverse effects on the heart (Guazzi and Borlaug, 2012).

Previous research has seldom addressed the cascade of cardiac complications triggered by VHDs. However, several studies, including one by Villari et al. (1992), suggest that conditions such as and AR can lead to secondary cardiovascular issues. In this study, 58 patients with AS and AR, experiencing pressure and volume overload respectively, were assessed. The findings revealed that 50% of patients with AS and over 90% of those with AR (20 out of 22) exhibited abnormal diastolic function, despite maintaining a normal ejection fraction.

**FIGURE 4:**
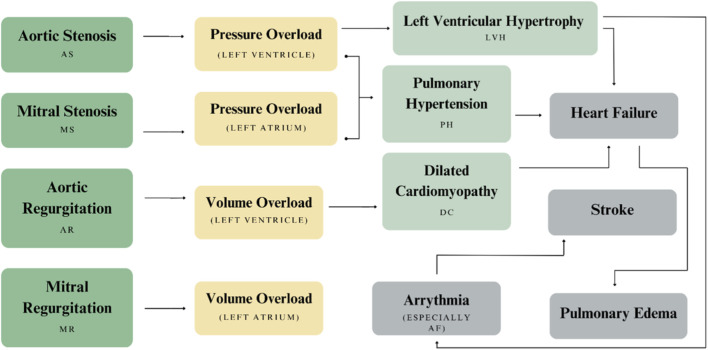
Clinical progression from VHDs to broader cardiovascular conditions.

As illustrated in Figure 4, the abnormal pressure and volume dynamics resulting from AS or AR can contribute to more severe CVDs, including Left Ventricular Hypertrophy (LVH), Type 2 Pulmonary Hypertension (PH), and Dilated Cardiomyopathy (Maeder et al., 2022; Schnelle, 2016; Schultheiss et al., 2019). If left untreated, these conditions can progress to heart failure (Heidenreich et al., 2022; Li et al., 2024), compounding the global burden of cardiovascular-related mortality. LVH, in particular, poses a significant risk if undiagnosed. As Bornstein et al. (2025), noted, “*patients with LVH remain asymptomatic for a few years*”, which often delays diagnosis until the condition advances to end-stage heart failure. In its later stages, LVH can also trigger arrhythmias, setting off a damaging chain of cardiac events that are difficult to reverse. These challenges emphasize the critical need for solutions that can effectively treat VHDs in their early stages, thereby preventing the onset of more serious complications (Waghmare and Zeba Khannam, 2024).

## 3 Patient specific approaches

### 3.1 Principles and benefits of personalized medicine in heart valve engineering

Personalized medicine, also known as precision medicine, is a therapeutic approach that tailors treatment to an individual’s unique genomic and proteomic profile, with the goal of preventing or eradicating disease (Beneduce and Bertolaso, 2022). Although its most notable applications have been in oncology, particularly for cancer patients, (Waghmare and Zeba Khannam, 2024; Druker et al., 2001), the potential of personalized medicine extends far beyond cancer care. Given the heterogeneous nature of cardiovascular diseases (CVDs), including valvular heart diseases (VHDs), personalized approaches could prove revolutionary in advancing cardiac care.

The benefits of precision medicine have been explored in earlier studies; however, research has accelerated significantly over the past decade. One key advantage is the improved risk-benefit ratio of treatment - favoring increased benefits - since therapies are tailored to each individual’s molecular profile (Baird et al., 2023). Personalized therapy is especially crucial in age-related conditions, particularly in pediatric patients. As noted by Kwon *et al* (Kwon et al., 2021), conventional treatments such as mechanical and bioprosthetic valves cannot grow alongside a developing heart, thereby necessitating additional interventions (Kwon et al., 2021). These repeated procedures contribute to rising healthcare costs, adding to the already substantial estimated annual expenditure of approximately $56.62 billion in the United States (Mallow et al., 2019).

An essential component of precision medicine - particularly in cardiology - is cell sourcing. In tissue engineering, regenerative and precision medicine intersect, and cells are selected based on the specific tissue being engineered. As discussed by Guilak *et al.*, these cells may be autologous (harvested from the patient) or allogeneic (sourced from a donor). Multipotent or pluripotent stem cells are often chosen for this process due to their ability to differentiate into nearly any cell type in the human body (Guilak et al., 2014). Techniques for efficiently using these cells to repair or regenerate organs and tissues are advancing rapidly (Liu et al., 2022). However, research in this area also faces regulatory and ethical barriers in several countries.

One of the most debated topics involves human embryonic stem cells (hESCs). While ethical concerns surrounding hESCs are still being explored due to the relative novelty of the field, a comprehensive review by Volarevic et al. (2018), outlines that nuclear transfer (NT) is prohibited in many countries, even though the use of hESCs in both *in vivo* and *in vitro* settings is permitted*.* Furthermore, hESCs may lead to tumor formation, posing significant clinical risks. These stem cells can become “*difficult to control after in vivo transplantation*” (Volarevic et al., 2018).

### 3.2 Patient-specific cell types for heart valve bioengineering: methods and applications

Several methodologies exist for procuring valvular cells, each offering distinct advantages for the bioengineering of heart valves. An overview of the cell types considered in this work is provided in Table 1.

**TABLE 1:** Stem cells, their advantages and disadvantages.

Cell type	Differentiation potential	Source	Key advantages	Key challenges	References
Induced Pluripotent Stem Cells (iPSCs)	Pluripotent (can differentiate into any cell type)	Adult somatic cells (e.g., skin fibroblasts)	- Autologous use reduces immune rejection - Circumvents ethical issues of hESCs - Can be expanded *in vitro* for large-scale production	- Low isolation yield from adult tissues - Risk of tumorigenicity (teratoma formation) - Genetic mutations accumulate during culture	(Caetano et al., 2004; Kögler et al., 2004; Yasuda et al., 2018; Lezmi and Benvenisty, 2022)
Mesenchymal Stem Cells (MSCs)	Multipotent (can differentiate into fibroblasts, osteoblasts, chondrocytes, adipocytes)	Bone marrow, adipose tissue, umbilical cord blood	- Promote ECM formation (collagen, elastin, glycosaminoglycans) - Enhance angiogenesis *via* VEGF secretion - Umbilical cord blood-derived MSCs avoid ethical concerns	- Ethical concerns over donor consent and commercialization - Limited differentiation potential compared to iPSCs - Regulatory challenges due to treatment novelty	(Parvin Nejad et al., 2023; Poomani et al., 2024; Seow et al., 2023)
Endothelial Progenitor Cells (EPCs)	Progenitor (can differentiate into endothelial cells)	Bone marrow, circulating bloodstream	- Enhance vascularization in engineered heart valves - Improve graft integration and structural stability	- Genetic and epigenetic instability - Require regular genetic stability verification	(Rashidi et al., 2024; Nikolskii et al., 2007)

#### 3.2.1 Induced pluripotent stem cells

For patient-specific applications, autologous induced pluripotent stem cells (iPSCs) derived from the patient are crucial to eliminate the risk of immune rejection. iPSCs are pluripotent, meaning they can differentiate into any cell type in the body. Additionally, they avoid the ethical and logistical concerns associated with human embryonic stem cells (hESCs), as iPSCs are generated from adult somatic cells rather than embryonic sources (Amabile and Meissner, 2009).

Adult somatic cells, which are non-reproductive, play a key role in maintaining tissue homeostasis by replacing senescent or damaged cells. However, their differentiation potential is limited to producing cell types specific to their tissue of origin. This limitation is overcome by reprogramming adult somatic cells into induced pluripotent stem cells (iPSCs). The most commonly used method involves introducing specific transcription factors (Oct4, Sox2, Klf4, and c-Myc) to initiate cellular reprogramming, sometimes with the aid of plasmid transfection (Caetano et al., 2004) (Kögler et al., 2004). Once reprogrammed, iPSCs regain pluripotency, enabling them to differentiate into virtually any cell type. By guiding this differentiation, researchers can generate substantial quantities of target cells, which are then seeded onto biocompatible scaffolds to construct implants or tissue constructs for tissue engineering (Rančić et al., 2020) (Li et al., 2017).

**FIGURE 5:**
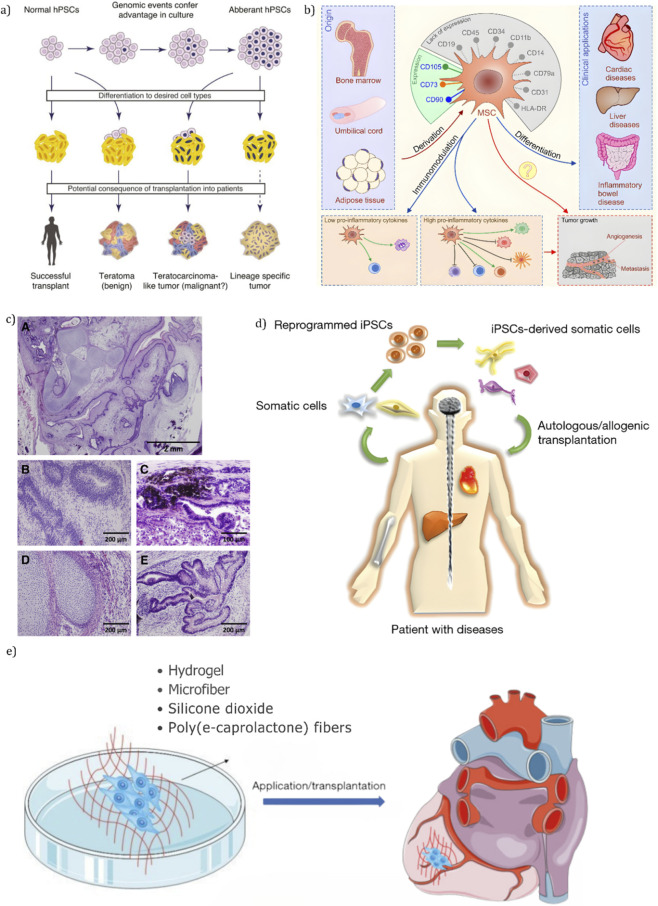
Stem cell-based strategies for cardiovascular repair and their potential risks. **(a)** Genetic instability in cultured iPSCs may lead to teratoma or lineage-restricted tumors upon differentiation and transplantation (Lezmi and Benvenisty, 2022). **(b)** During transplant, MSCs obtained from bone marrow, umbilical cord, or fat contain immunomodulatory properties and can promote repair in cardiac or inflammatory diseases (Seow et al., 2023). **(c)** Histopathological sections of iPSC-derived teratomas serves as examples of different tissue types as somatic cell-derived stem cells indicating risks for tumorigenesis (Yasuda et al., 2018). **(d)** iPSCs can used to create patient-specific somatic cells for either autologous or allogeneic tranplant into populations of patients with degenerative diseases (Li et al., 2017). **(e)** Biomaterial scaffolds-i.e., hydrogels, polycaprolactone fibers, enhances stem cell delivery, and can take the form of scaffold for implantation into engineered heart valves (Dandeniyage et al., 2019).

Nonetheless, there are limitations to this approach. A significant challenge is the consistently low yield of adult somatic cells during isolation from their native tissues, which negatively impacts the efficiency of iPSC generation (Caetano et al., 2004) (Kögler et al., 2004). Another limitation is the tumorigenicity of iPSCs. As captured in Figure 5c, during *in vivo* transplantation, undifferentiated stem cells can give rise to teratomas (Yasuda et al., 2018). This occurs because pluripotent stem cells tend to accumulate genetic mutations during *in vitro* culture. For example, a study by Lezmi and Benvenisty (2022) revealed an average of about five non-synonymous mutations per cell line, as shown schematically in Figure 5c (Lezmi and Benvenisty, 2022).

However, there is potential to mitigate risks of tumorigenicity. iPSCs can become tumorigenic because they share part of their gene expression profiles with cancer cells (Zhang et al., 2015). Therefore it is possible to use either an antibody-cytotoxic drug conjugates, (designed to target a unique antigen on iPSCs) (Sougawa, 2016) or genetic reprogramming and reducing the use of oncogenic factors in iPSCs (Lin et al., 2024).

#### 3.2.2 Mesenchymal stem cells

Mesenchymal Stem Cells are multipotent cells capable of differentiating into various cell types, such as osteoblasts, chondrocytes, and adipocytes. Figure 5b exhibits how they can be derived from multiple sources, being most commonly derived from sources such as bone marrow, adipose tissue, and umbilical cord blood. In heart valve tissue engineering (HVTE), MSCs are particularly valuable due to their ability to mimic the fibroblast subpopulation found in native heart valves and their involvement in myofibrogenesis - crucial for producing key extracellular matrix (ECM) components including collagen, elastin, and glycosaminoglycans within the valve leaflet, as discussed by Parvin Nejad et al. (2023).

Poomani et al. (2024) further highlight their reparative properties, such as the promotion of angiogenesis through the release of growth factors like vascular endothelial growth factor (VEGF) (Poomani et al., 2024) However, ethical concerns related to MSC use persist. These include the need for informed donor consent, prevention of exploitation, regulation of commercialization to ensure equity, safeguarding patient safety, and addressing the use of animal models in research. Standardized regulatory frameworks are essential to address these concerns but remain under development due to the relative novelty of the therapy. Despite these challenges, MSCs show significant promise. As shown in Figures 5b,d, MSCs contain a diverse range of exosomes with distinct capabilities determined by genetic factors, which further enhances their therapeutic potential (Seow et al., 2023).

A particularly abundant and ethically favorable source of MSCs is umbilical cord blood. It offers a rich supply of MSCs without ethical drawbacks, as its collection poses no harm to either the mother or the child, and the umbilical cord is typically discarded. Furthermore, these stem cells can be cryopreserved for extended periods, making them a valuable and accessible resource for regenerative medicine.

#### 3.2.3 Endothelial progenitor cells

Endothelial progenitor cells (EPCs) are stem cells that can become endothelial cells, playing a key role in blood vessel formation and tissue repair. Sourced from bone marrow, they circulate in the bloodstream and migrate to areas of injury or damage to support vascular repair and regeneration.

The incorporation of endothelial progenitor cells (EPCs) into engineered vascular grafts can improve both the structural integrity and overall functionality of bioengineered heart valves.

By encouraging the formation of new blood vessels within these grafts, EPCs help create a supportive environment for the valves, which in turn enhances their integration into the host tissue. This vascularization not only strengthens the valve but also ensures better long-term function, facilitating smoother incorporation into the patient’s circulatory system (Rashidi et al., 2024).

However, research has demonstrated that numerous human embryonic stem cell (ESC) lines experience genetic and epigenetic alterations over time, potentially impacting their suitability for both research and therapeutic purposes. For example, it has been noted that the genetic stability of EPC lines requires regular verification, as many lines display changes that could result in malignant transformations (Nikolskii et al., 2007).

### 3.3 Role of genetic and phenotypic profiling in treatment customization

Building upon the use of patient-specific cells, genetic and phenotypic profiling plays a critical role in fine-tuning heart valve therapies to match the unique needs of each individual. Research on the importance of genetic and phenotypic profiling has been done.

Profiling has been essential in prevention of Graft-versus-host disease (GVHD). GHVD remains a critical challenge in allogeneic hematopoietic cell transplantation (HCT). Early studies emphasized the role of immune dysregulation, particularly within T-cell subsets, at the onset of acute GVHD (aGVHD). Cellular and molecular profiling of T cells has revealed distinct changes in immune responses, such as a reduction in native T cells and an expansion of effector memory T cells. These findings highlight the critical shifts in T-cell homeostasis during the disease process and suggest potential avenues for targeted therapies (Latis et al., 2019). Furthermore, molecular profiling has implicated pathways such as decreased TGF-β signaling and increased NF-kB activity in the pathogenesis of GVHD, offering insights into the complex interplay of signaling mechanisms that drive the condition (Latis et al., 2019).

Gene-expression profiling of donor T cells has also emerged as a promising tool for predicting GVHD occurrence in transplant recipients. By analyzing gene interactions and expression levels, researchers identified specific gene pairs that distinguish between GVHD-positive and GVHD-negative cases, with high predictive accuracy. These discoveries not only provide a mechanism for donor risk stratification but also underscore the potential of gene profiling in improving transplant outcomes (Amabile and Meissner, 2009). The predictive capability of gene-expression profiling is further supported by studies employing advanced statistical methods, such as linear discriminant analysis and predictive interaction analysis, to assess gene interactions. These approaches have significantly enhanced the precision of GVHD prediction models, facilitating better donor selection and personalized treatment strategies (Caetano et al., 2004).

The integration of T-cell subset dynamics, gene-expression profiling, and molecular pathway analysis represents a transformative approach to understanding and managing GVHD. This multi-faceted methodology not only addresses the heterogeneity of immune responses in transplantation but also paves the way for innovative therapies that could mitigate GVHD-related complications and improve patient survival rates (Latis et al., 2019; Socie and Michonneau, 2022; Baron et al., 2007). A similar method is utilized in the field of cancer immunotherapy, where early therapies targeted broad immune responses. As discussed by Sun et al. (2024), by incorporating genetic and phenotypic profiling, treatments like CAR-T cell therapy have achieved more precise targeting of cancer cells, significantly improving patient outcomes. In this case, genetic profiling was used to identify tumor-specific antigens, while phenotypic profiling helped tailor immune responses to individual patient needs (Sun et al., 2024).

## 4 Immunomodulation techniques

### 4.1 Strategies to enhance graft acceptance and reduce immune response

Graft acceptance remains a significant challenge in transplantation and tissue engineering. Although studies on cardiac bioengineered graft transplantation remain scarce, a recent investigation by Gutowski et al. on HAV revealed a gradual decline in graft functionality over time, with primary patency rates decreasing to 44% at 72 months (Gutowski et al., 2022). These findings highlight the need for further research into the immunological factors affecting HAV integration and long-term performance. To address this issue, three primary strategies have emerged: biomaterial-based immunoengineering, MHC matching, and immunosuppressive therapies. Table 2 compares the immunomodulatory techniques examined in this study.

**TABLE 2:** Immunomodulatory techniques, their advantages and disadvantages.

Strategy	Mechanism	Advantages	Challenges	Relevant citations
Biomaterial-Based Immunoengineering	Uses 3D platforms, hydrogels, and nanoparticles to deliver localized immunosuppressive therapies	- Localized immune modulation, reducing systemic side effects - ECM-mimicking biomaterials enhance graft integration	- Requires optimized biomaterial selection for long-term immune regulation - Scalability issues for clinical applications	(Abbaszadeh et al., 2023; Nadine et al., 2021; Ma et al., 2024)
3D Immunomodulatory Platforms	Engineered biomaterials release immunosuppressive agents in a controlled manner	- Targeted delivery reduces rejection risk - Enhances tissue integration through controlled cytokine release	- Limited clinical validation for heart valve applications - Material degradation rates vary, affecting long-term function	(Nadine et al., 2021; Ma et al., 2024)
Nanoparticle Vaccines	Engineered particles deliver immune-modulating agents to induce tolerance	- Stimuli-responsive release ensures controlled drug delivery - Induces immune tolerance by increasing regulatory T cells (Tregs)	- Potential systemic side effects such as cytokine release syndrome - Long-term safety concerns in clinical applications	(Meng et al., 2024; Hristova-Panusheva et al., 2024; Huang et al., 2022)
MHC Matching	Aligns donor and recipient MHC molecules to reduce immune rejection	- Prevents graft rejection without heavy reliance on immunosuppressants - Preserves immune function while allowing tolerance	- Requires precise genetic screening before transplantation - Not feasible for all patients due to donor availability	(George et al., 2019; Dalheimer et al., 2005a; Shanmugarajah et al., 2017)
Immunosuppressive Drug Therapies	Suppresses immune activation through T-cell inhibition, cytokine modulation, and proliferation suppression	- Proven clinical efficacy in organ transplantation - Prevents acute rejection, improving graft longevity	- High toxicity risks (e.g., cardiovascular effects, infections) - Potential long-term metabolic disturbances	(Claeys et al., 2019; Romary et al., 2024; Miller, 2002)

#### 4.1.1 Biomaterial-based immunoengineering

The first approach, biomaterial-based immunoengineering, includes 3D immunomodulatory platforms - engineered systems that deliver immunosuppressive therapies directly to the transplantation site. These biocompatible platforms enable controlled, sustained release of agents such as drugs or bioactive molecules, enhancing therapeutic precision while minimizing systemic side effects. Their 3D architecture allows spatial and temporal control of delivery, supporting localized immune modulation and promoting graft tolerance. By mimicking the extracellular matrix, they also create a favorable microenvironment for graft integration. Overall, these localized strategies offer a safer, more targeted alternative to systemic immunosuppression, reducing both immune rejection and adverse effects (Abbaszadeh et al., 2023; Nadine et al., 2021).

**FIGURE 6:**
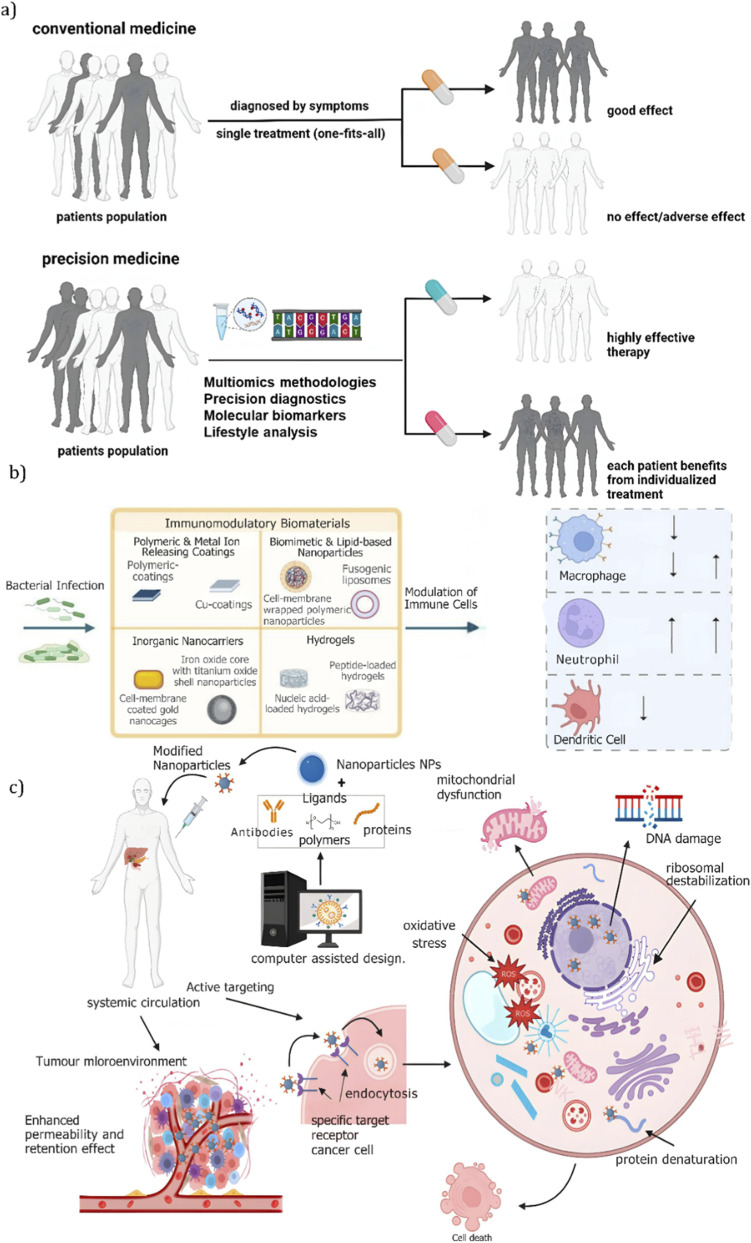
Translational strategies in precision medicine and nanotherapeutics. **(a)** Precision medicine uses molecular diagnostics to tailor treatments, improving outcomes compared to conventional one-size-fits-all approaches (Hristova-Panusheva et al., 2024). **(b)** Immunomodulatory biomaterials - such as coatings, hydrogels, and nanoparticles - modulate innate immune responses to combat infection (Gomez Casas and Shukla, 2024). **(c)** Nanoparticles enable targeted drug delivery to tumors, inducing intracellular stress and selective cancer cell death (Hristova-Panusheva et al., 2024).

3D immunomodulatory platforms utilize diverse materials, with each study showcasing unique applications. Nadine et al. (2021) introduced liquefied capsule-based platforms to evaluate polymers like poly (L-lysine), alginate, and chitosan. In their proof-of-concept study, capsules with chitosan and encapsulated mesenchymal stem/stromal cells (MSCs) were placed on macrophage cultures, where they promoted a regenerative immune response by upregulating anti-inflammatory markers and releasing pro-regenerative cytokines (Nadine et al., 2021). Kim et al. however, explored natural biomaterials such as silk, collagen, and fibrin, emphasizing their use in creating bioengineered scaffolds for tissue integration. Table 3 presents the hemocompatibility and biocompatibility profiles of the materials examined in this study. The study highlighted these materials’ biocompatibility and ease of surface functionalization, allowing them to modulate immune responses while minimizing toxicity, particularly in applications requiring extracellular matrix mimicry (Ma et al., 2024). Casas and Shukla investigated immunomodulatory nanoparticles and hydrogels, focusing on their role in controlled drug delivery. Figure 6b demonstrates that lipid-based and biomimetic nanoparticles effectively delivered immune-modulating agents, while hydrogels provided localized immune control in wound healing models, reducing inflammation and supporting tissue regeneration (Gomez Casas and Shukla, 2024). Overall, polymers like chitosan, natural biomaterials such as collagen and fibrin, and advanced systems like nanoparticles and hydrogels enable targeted immune modulation and localized therapy, supporting tissue integration, graft survival, and regeneration in transplantation.

**TABLE 3:** Comparison of scaffold materials key properties and concerns.

Material/System	Strength/Mechanics	Bio/Hemocompatibility	Main concern	Citation
Silicone polyurethanes	Lower strength, durability concern	Reduced cell/protein adhesion, less clotting	May not tolerate long-term load	Liu et al. (2024)
SiPUU	High tensile and tear strength	Elastomeric behavior	Inconsistent mechanics	Dandeniyage et al. (2019)
LifePolymer	Stable in testing	Low thrombogenicity, good biostability	Long-term structural integrity unclear	Jenney et al. (2021)
POSS-PCU	Withstands physiologic stress in vitro	Resists calcification vs. pericardium	Limited long-term in vivo data	Ghanbari et al. (2010)
Hastalex	Stronger than GORE-TEX	Hemocompatible, hydrophilic surface	Fatigue life not proven	Ovcharenko et al. (2020)
PCL/PGS/PSf scaffold	Native-like anisotropy, aligned fibers	Supports cellular organization	Little in vivo data, scale-up issues	Gürbüz et al. (2024)
SIBS-MWCNT	Higher tensile strength	Improved hydrophilicity	Sensitive to nanotube loading	(Rezvova et al., 2023)
PVDF/PCU/SEPS/SEBS	High tensile strength; long-cycle survival; near ISO 5840	Good hemodynamics, less calcification	PVDF stiffness; needs long-term fatigue data	(Boehm et al., 2023); (Stasiak et al., 2020)

The second method involves nanoparticle vaccines - engineered particles designed to prevent immune rejection of grafts by delivering antigens or immunosuppressive agents. Several experimental studies indicate that these nanoparticles help induce immune tolerance, suppress inflammation, and target key immune cells. As discussed in a comprehensive review by Meng et al. (2024), recent advances have enabled more precise delivery, with nanoparticles engineered to release their payload only in response to specific stimuli, such as pH or temperature changes. Hristova-Panusheva et al. (2024) further highlight that nanoparticles can be functionalized to target specific cells *via* ligand–receptor interactions, enabling receptor-mediated endocytosis for efficient drug delivery. Stimuli-responsive systems thus allow controlled release in targeted microenvironments. In terms of contents*,* the Hristova-Panusheva et al. study emphasized how nanovaccines containing IL-2 and TGF-β increase regulatory T cells (Tregs), which are key for suppressing immune overreactions. Tregs reduce alloreactivity (immune attack on foreign cells) and promote immune tolerance, creating a balanced, tolerogenic environment that prevents rejection and supports immune regulation. The study found that treatment with the tolerogenic nanoparticles (NPs) resulted in a marked inhibition of mixed lymphocyte reaction (MLR) to donor cell alloantigen. As evidenced in the visual Figures 6a,c, this indicates a significant shift from an immunogenic response to a tolerogenic one (Hristova-Panusheva et al., 2024). Most of these studies, however, are based on preclinical models, and require further *in vivo* testing.

Although nano-immunotherapy presents promising advancements for hematological malignancies; however, it comes with notable challenges and adverse effects. Immune-related adverse effects (irAEs), such as cytokine release syndrome (CRS) and neurotoxicity, can result in systemic inflammation and neurological complications, requiring careful monitoring and timely intervention (Huang et al., 2022). Additionally, systemic inflammation triggered by immune activation may extend beyond the tumor microenvironment, affecting healthy tissues and organs. This inflammatory response increases the risk of organ dysfunction, including hepatic, renal, or cardiac impairment, which necessitates vigilant observation and tailored management strategies.

To address these challenges, further refinements in nanotechnology are essential to improve immune targeting, minimize off-target effects, and ensure safer clinical applications of nano-immunotherapies.

#### 4.1.2 Major histocompatibility complex (MHC) matching

The Major Histocompatibility Complex (MHC) is fundamental in transplantation immunology, as mismatches between donor and recipient MHC molecules often lead to rejection. However, recent advances have demonstrated strategies to bypass this barrier. George et al. (2019), Dalheimer et al. (2005a) explored a novel conditioning regimen involving six monoclonal antibodies that suppress hematopoietic stem cells (HSCs), T cells, and NK cells without requiring radiation or chemotherapy. This breakthrough allowed MHC-mismatched HSC transplantation while inducing immune tolerance, enabling recipients to accept solid organ grafts from the same donor.

A key immunological finding of this approach is that tolerance does not compromise overall immunity. The study revealed that recipient animals retained their ability to generate antibody responses to new antigens, demonstrating that immune functionality remains intact despite the induced tolerance (Shanmugarajah et al., 2017). This preservation of functional immunity is particularly relevant for patient-specific treatments, where minimizing immune suppression without weakening defenses is a clinical priority.

Further immunological insights focus on how MHC compatibility influences graft infiltration. Dalheimer et al. demonstrated that when both donor and host share class I MHC alleles, there is enhanced infiltration of grafts by mHAg-specific CD8 T cells, which play a central role in graft surveillance and rejection (Dalheimer et al., 2005b). This highlights the importance of MHC presentation in modulating immune responses and suggests that minor histocompatibility antigen (mHAg) interactions must be carefully managed to reduce rejection risks.

Collectively, these studies underscore the critical role of MHC and immunoregulation mechanisms in transplantation. These findings pave the way for bioengineered heart valves and other patient-specific therapies, where reducing rejection risk without extensive immunosuppression is essential.

**FIGURE 7:**
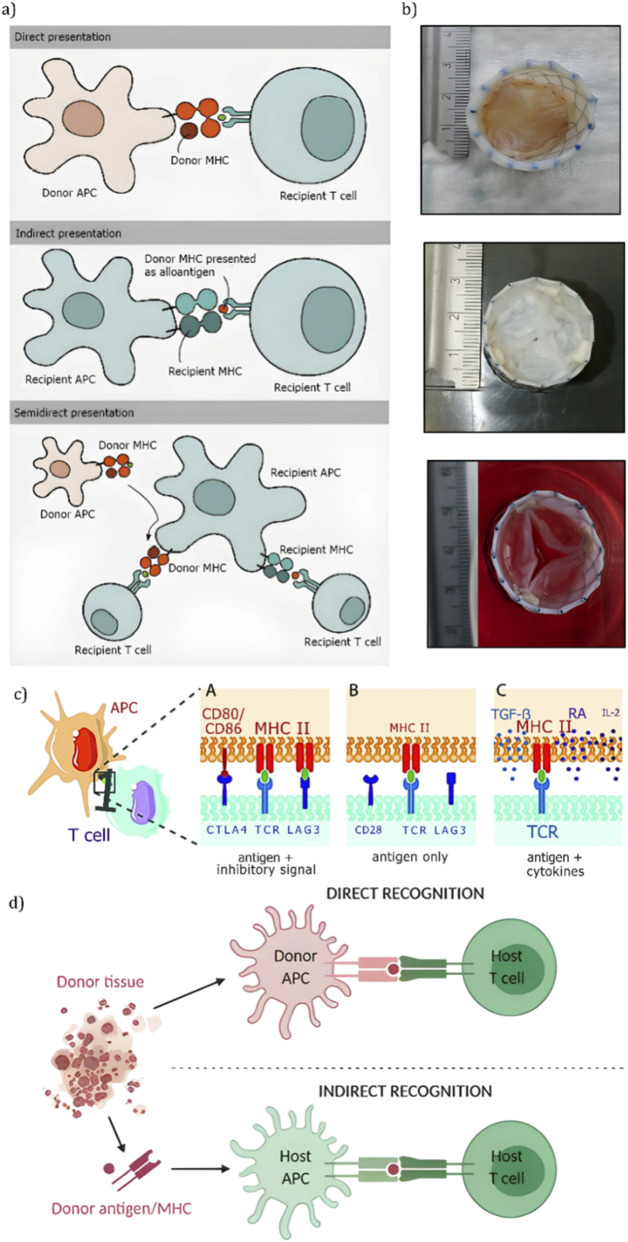
Overview of alloantigen recognition pathways and immunoregulatory signals in transplantation. Overview of alloantigen recognition pathways and immunoregulatory strategies in transplantation. **(a)** Pathways of direct, indirect, and semidirect antigen presentation from donor to recipient T cells (Dalheimer et al., 2005b). **(b)** Representative decellularized porcine heart valve scaffolds prepared for xenograft applications (Saeid Nia et al., 2024). **(c)** T cell activation at the APC interface involves antigen recognition, co-stimulatory/inhibitory signaling, and cytokine-mediated modulation (Emerson et al., 2020). **(d)** Simplified model of direct and indirect T cell recognition of donor antigens in graft rejection (Claeys et al., 2019).

As discussed in a comprehensive review by Claeys and Vermeire (Claeys et al., 2019), immunosuppressive drugs target various immune pathways to prevent organ rejection. Agents like antithymocyte globulin (ATG) and Alemtuzumab deplete both T and B cells, while Rituximab specifically targets B cells. As illustrated in Figure 7d, Basiliximab and Belatacept inhibit T cell activation. Proliferation inhibitors such as Azathioprine and mycophenolate mofetil (MMF) suppress immune cell growth, whereas calcineurin inhibitors (e.g., Cyclosporine and Tacrolimus) reduce cytokine production. Sirolimus and Everolimus inhibit the mTOR pathway, and corticosteroids broadly suppress immune activity. While these drugs are critical for preventing rejection and improving graft survival, they are associated with significant side effects, including toxicity, increased infection risk, and metabolic disturbances - factors that complicate long-term patient outcomes.

#### 4.1.3 Immunosuppressive drugs

A recent study by Romary et al. (2024) examined the impact of systemic immunosuppression on revascularization outcomes in patients with chronic limb-threatening ischemia (CLTI). Although these drugs are essential for managing immune responses, concerns persist about their potential to impair vascular healing. The study compared perioperative outcomes, reintervention rates, and long-term patency between immunosuppressed and non-immunosuppressed patients undergoing infrainguinal bypass grafting (BPG) or percutaneous transluminal angioplasty with stenting (PTAS). Interestingly, the results revealed no significant differences between the groups, suggesting that systemic immunosuppression does not adversely affect revascularization success. These findings challenge long-held assumptions about the negative vascular effects of immunosuppressants and underscore the need for further investigation into specific drug classes and patient subgroups (Romary et al., 2024).

Miller’s (Miller, 2002) review raises another key concern: the cardiovascular toxicity of immunosuppressive agents. While drugs such as Cyclosporine, Tacrolimus, and Rapamycin offer effective immunosuppression, they have been linked to hypertension, dyslipidemia, and accelerated atherosclerosis, all of which heighten cardiovascular disease risk in transplant patients. Lipid-lowering therapies like pravastatin and fenofibrate have shown promise in mitigating these risks; however, drug–drug interactions - particularly with calcineurin inhibitors - remain a challenge. Despite advancements in therapy, the long-term toxicity of immunosuppressants continues to affect both patient survival and graft longevity. Miller’s findings emphasize the importance of cardiovascular monitoring and the development of newer, safer immunosuppressive agents. However, as the review is over 2 decades old, its conclusions should be revisited in the context of contemporary treatment regimens.

### 4.2 Surface modifications and biomaterial strategies for immune tolerance in bioengineered heart valves

Biomaterial-based approaches play a key role in modifying cell or material surfaces to develop tolerogenic therapies. A notable example is antigen coupling to cell surfaces using ethylene carbodiimide (ECDI) chemistry, which has shown significant effectiveness in inducing tolerance across various mouse models, including those for allergy, transplantation, and autoimmune diseases. As highlighted by Emerson et al. (2020), and as visually narrated in Figure 7c, these findings represent a paradigm shift in immunotherapy, demonstrating how biomaterials can be precisely engineered to reprogram immune responses.

Biomaterials are also utilized to deliver tolerogenic biologics, drugs, or antigens in a controlled and sustained manner. The delivery of cytokines, such as TGF-β1, has shown profound effects in maintaining regulatory immune states and inducing T regulatory cells (Tregs), particularly in type 1 diabetes models (Emerson et al., 2020). These breakthroughs exemplify the versatility of biomaterial platforms in fostering immunological tolerance while minimizing systemic side effects - a key step forward in advancing precision medicine.

Heart valve replacement remains a cornerstone intervention for valvular heart disease, yet its challenges are particularly pronounced in pediatric patients due to growth constraints and durability concerns. A study by Nia et al. introduced an innovative decellularization protocol that preserves the extracellular matrix (ECM) structure while minimizing residual DNA, effectively reducing immunogenicity risks (Saeid Nia et al., 2024). Their thorough analysis of DNA content and the mechanical properties of decellularized heart valves underscores the clinical importance of maintaining ECM integrity for long-term graft functionality. Earlier studies, such as those by Rieder et al., provided foundational insights, though evolving techniques now offer improvements over previous methods (Rieder et al., 2004).

To address the persistent issue of calcification in bioprosthetic heart valves, a dual approach combining decellularization with engineered crosslinking has emerged as a promising solution. This method not only enhances durability but also mitigates immunogenicity and calcification risks - two critical barriers to clinical success (Human et al., 2020). Liu et al. demonstrated that enhanced crosslinking significantly reduced calcification in bovine and porcine pericardium models following implantation in rats (Liu et al., 2024). While longer-term studies are needed, this approach could pave the way for more resilient and longer-lasting bioprosthetic heart valves. Lídia et al. (2021) developed a decellularization protocol for porcine hearts to create biological scaffolds for tissue engineering. Using 4% sodium dodecyl sulfate (SDS) and 1% Triton X-100, the process effectively removed cells while preserving key ECM components like collagen, elastin, and glycosaminoglycans (Lídia et al., 2021). The decellularized scaffold retained its 3D structure and supported cell adhesion and proliferation during recellularization. Both microscopic and macroscopic analyses confirmed minimal morphological changes, highlighting the protocol’s potential for heart bioengineering and scaffold biocompatibility (Liu et al., 2024).

These types of biomaterial-based strategies can be divided into two categories; localized (implant site, nano-immunotherapy, surface modifications) and systemic (immunosuppressants). Each has its specific drawbacks and risks that must be accounted for. Localized therapies have stark lack of clinical validation for heart valves; which can be hazardous due to the uniquely challenging environment heart valves must be equipped for (Latis et al., 2019; Socie and Michonneau, 2022) Furthermore, nano therapy specifically often causes immune-related adverse effects (irAEs), such as cytokine release syndrome (CRS) and neurotoxicity (Abbaszadeh et al., 2023; Nadine et al., 2021) Systemic strategies have been tested more thoroughly *in vivo*; but are also associated with similar issues of cardiovascular toxicity. They also slow down endothelial cell proliferation, which can have adverse effects on healing (Dalheimer et al., 2005a; Shanmugarajah et al., 2017) Both have their strengths and drawbacks; which underscores the importance of patient-specific treatment along with advancement to overcome these toxicological issues.

Biomaterial-based strategies, by improving durability, reducing immunogenicity, and enhancing biocompatibility, have shown promise. However, it is important to continue further research on their long-term biological stability, and manufacturability.

## 5 Bioengineering techniques

### 5.1 Selection criteria for scaffold materials in heart valves

#### 5.1.1 Proven performance and biocompatibility

Materials such as silicone polyurethanes, polycarbonate urethanes, and nanocomposite polymers have proven essential in addressing the performance and biocompatibility challenges of artificial heart valves. However, despite their suitability for medical applications, their use presents potential drawbacks that require careful consideration. A critical evaluation of their mechanical and biological properties reveals limitations that could affect their long-term efficacy, especially in high-stress environments like the heart.

**FIGURE 8:**
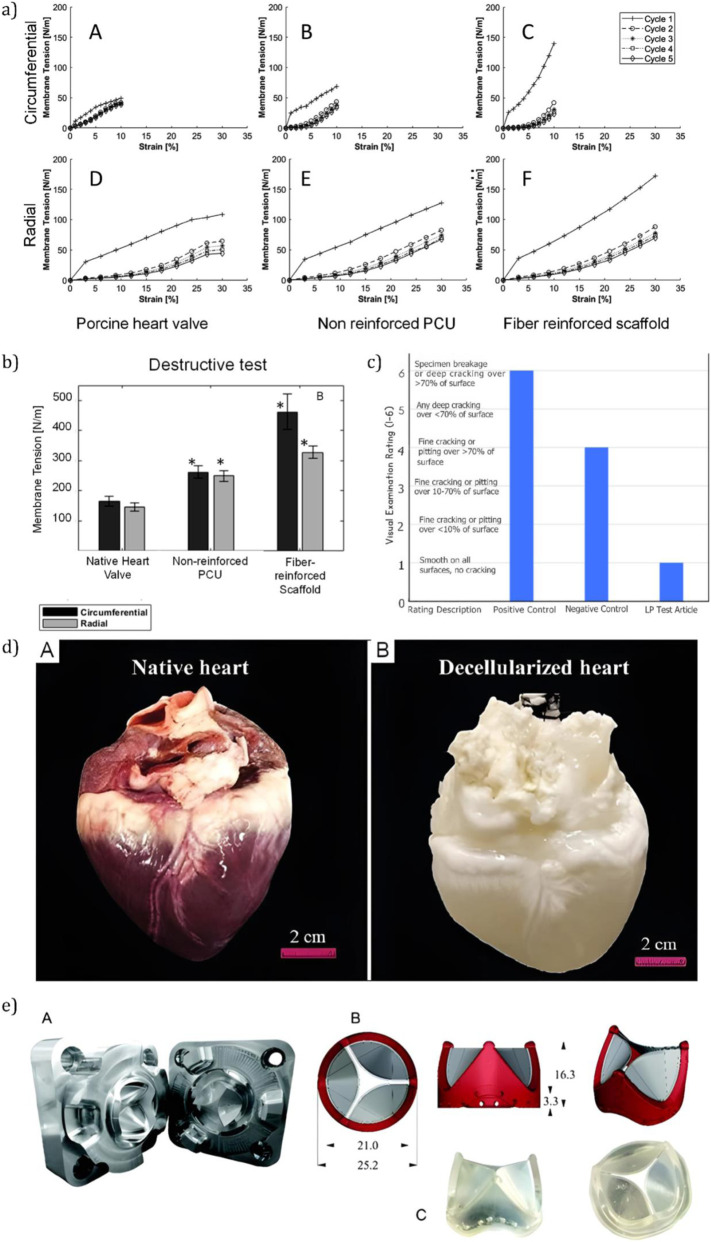
Patient specific biomaterials for heart valve engineering. **(a, b)** Biaxial tensile testing of electrospun polycarbonate urethane (PCU) scaffolds shows that fiber-reinforced (PVDF fiber) scaffolds have dramatically higher anisotropic tensile strength and stiffness than both native porcine valve leaflets and non-reinforced PCU (Boehm et al., 2023). **(c)** A novel siloxane-based poly (urethane-urea) elastomer (LifePolymer™) for valve leaflets exhibits a low dynamic modulus, high tensile strength, and minimal creep - properties that together indicate robust mechanical performance and resistance to tearing under physiological loading (Jenney et al., 2021). **(d)** Gross images of whole porcine hearts before (native) and after detergent-based perfusion decellularization demonstrate complete cell removal (pale, translucent appearance) while preserving the overall cardiac architecture and ventricular volume (Lídia et al., 2021). **(e)** A tri-leaflet polymeric valve prototype (styrenic block copolymer) was fabricated by injection molding and bench-tested to ISO 5840 standards; it met durability benchmarks (>10^9^ fatigue cycles) and bioprosthetic-level hydrodynamic performance, and short-term *in vivo* (ovine) trials confirmed functional deployment without acute failure (Stasiak et al., 2020).

Silicone polyurethanes have demonstrated enhanced biocompatibility through the formation of silicon-enriched surface layers that reduce cell and protein adhesion, thereby mitigating risks of thrombosis. However, this improvement comes at the expense of mechanical strength, raising concerns about their durability in prolonged use (Liu et al., (Liu et al., 2024)). Similarly, siloxane poly (urethane-urea) elastomers (SiPUU) show favorable tensile and tear strengths, particularly when synthesized with PHMO macrodiols and specific diisocyanates, yet inconsistencies in mechanical performance and the absence of plastic deformation highlight the need for further refinement to ensure reliability (Dandeniyage et al., 2021) (Dandeniyage et al., 2019). LifePolymer™ (LP), another siloxane-based elastomer, has demonstrated minimal thrombogenicity and strong biostability, with the trend being apparent in Figure 8c, yet questions persist regarding its ability to maintain structural integrity over extended periods, as even slight variations in material properties could lead to functional failures Jenney et al. (2021). These findings suggest that while these materials offer significant advancements, their application in heart valve design requires a balanced approach that prioritizes both biocompatibility and mechanical resilience to meet the demands of cardiac function.

Nanocomposite polymers offer a distinct advantage in addressing the limitations of traditional polyurethane-based systems. For example, polyhedral oligomeric silsesquioxane-poly (carbonate-urea) urethane (POSS-PCU) has gained attention for its exceptional resistance to calcification - a major issue in biological valve replacements (Ghanbari et al., (Ghanbari et al., 2010)). Under *in vitro* conditions simulating physiological stress, this polymer outperforms conventional materials such as glutaraldehyde-fixed pericardium. However, despite its promising anti-calcification properties, the long-term structural performance of POSS-PCU and its integration with host tissues remain insufficiently studied, particularly under dynamic cardiac loading. Similarly, Hastalex - a functionalized graphene oxide-poly (carbonate-urea) urethane copolymer - has demonstrated superior mechanical strength and hemocompatibility compared to GORE-TEX (Ovcharenko et al., (Ovcharenko et al., 2020)). Although its hydrophilic surface and reduced calcific deposition are advantageous, concerns persist regarding its long-term fatigue resistance, which could affect clinical durability.

**FIGURE 9:**
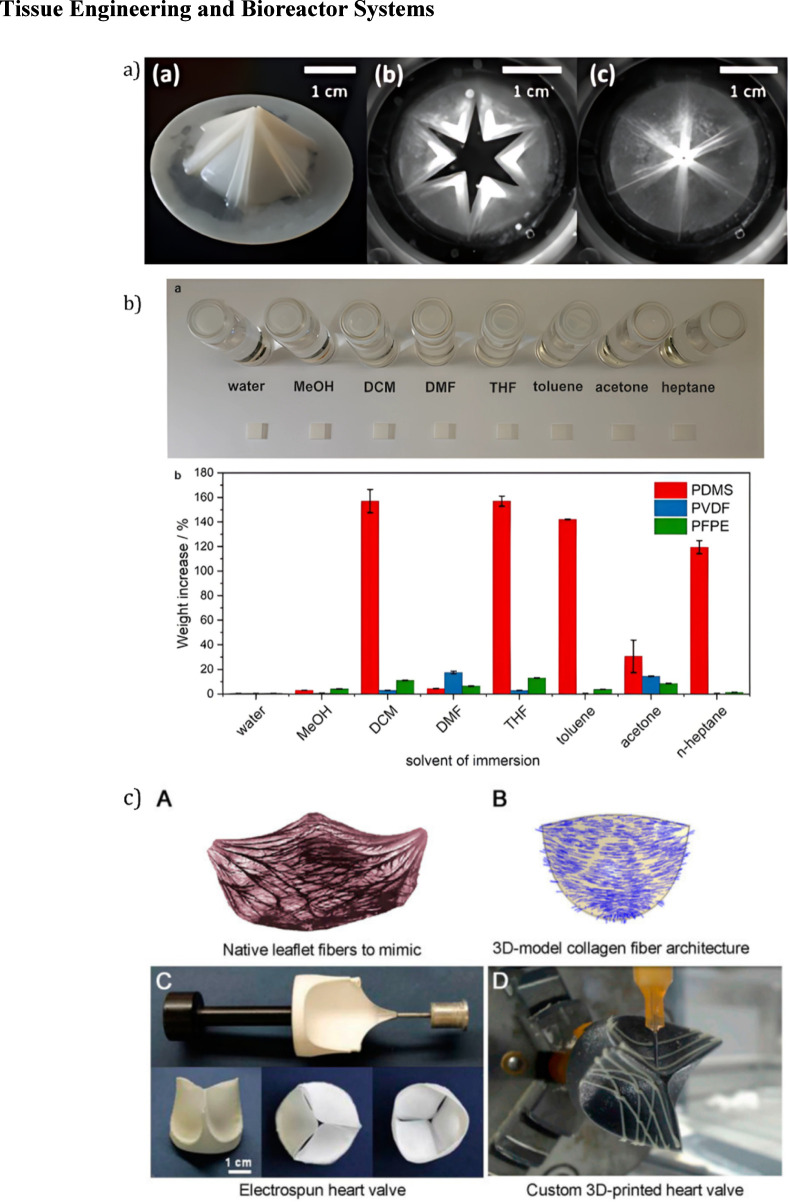
Additive manufacturing and materials for heart valve design. **(a)** Molded polymeric tri-leaflet heart valve prototypes demonstrating structural form and functional leaflet coaptation under simulated pressure (Schröter et al., 2023). **(b)** Solvent immersion and swelling test results showing weight change across PDMS, PVDF, and PFPE polymers, highlighting PVDF’s superior solvent resistance (Rein et al., 2024). **(c)** Native leaflet fiber orientation is mimicked using electrospun and custom 3D-printed heart valves incorporating controlled fiber alignment strategies (Rezvova et al., 2023).

Composite scaffolds such as the PCL/PGS/PSf system developed by Gürbüz et al. (2024) demonstrate promising advancements in replicating the native anisotropy and cellular alignment of the ventricularis layer. These features are critical for mimicking the structural and functional properties of natural heart valves. However, their translation to clinical application is currently hindered by limited *in vivo* data and challenges related to large-scale manufacturing. Similarly, SIBS-MWCNT nanocomposites introduced by Rezvova et al. (2023), visualized in Figure 9c, show enhanced tensile strength and improved hydrophilicity - key properties for withstanding hemodynamic stresses and supporting cellular integration. Nevertheless, the system’s sensitivity to nanotube concentration raises concerns regarding reproducibility and long-term performance. While both materials address vital shortcomings in traditional heart valve designs, the absence of extensive testing under physiological conditions underscores the necessity for continued preclinical development and validation.

In conclusion, conventional materials such as polyurethanes have been extensively studied and remain central to scaffold design in heart valve engineering. Their proven versatility, durability, and biocompatibility continue to address fundamental challenges, including resistance to calcification and mechanical fatigue. The emergence of nanocomposite polymers further enhances these properties, introducing novel possibilities for improving both structural integrity and biological interaction. However, the increasing complexity of these material systems underscores the importance of multidisciplinary collaboration across material science, biomechanics, and tissue engineering. Future research should focus on scalable fabrication techniques, comprehensive long-term *in vivo* assessments, and strategies to finely tune the interplay between mechanical performance and biological integration. Such efforts will be essential for translating these innovative materials into clinically viable, durable, and patient-specific heart valve replacements that meet the diverse functional demands of real-world applications.

#### 5.1.2 Mechanical properties

Mechanical strength is a critical factor in the design of tissue-engineered heart valves (TEHVs), as these constructs must endure the dynamic and repetitive mechanical stresses of the cardiovascular system while maintaining long-term functionality. Insights derived from the mechanical behavior of native heart valves, particularly their anisotropy, non-linear stress-strain relationships, and extracellular matrix (ECM) composition, play a crucial role in informing the development of biomimetic valve replacements. By closely replicating these structural and functional characteristics, researchers aim to engineer TEHVs that can match the resilience and performance of native valves, with particular importance in pediatric applications where growth and adaptability are essential.

As investigated by Nejad et al. (2024), the mechanical properties of pediatric pulmonary valves reveal several critical features that are essential for the design of effective tissue-engineered heart valves (TEHVs). Their anisotropic behavior, characterized through planar biaxial tensile testing, reflects the directional stress response of native valves and supports durability under the complex loading conditions of the cardiac cycle. The non-linear tension-strain relationship observed during testing further highlights their capacity to accommodate varying mechanical demands, with derived material constants serving as a foundation for predictive modeling aimed at optimizing TEHV performance. Moreover, the homogeneous distribution of extracellular matrix (ECM) components, organized into a trilayer structure primarily composed of collagen (60%), glycosaminoglycans (20%), and elastin (15%), ensures consistent mechanical integrity and mitigates localized stress concentrations, thereby reducing the risk of structural failure. Collectively, these findings offer valuable guidance for engineering TEHVs that closely replicate the mechanical functionality of native valves, with relevance to pediatric applications where long-term durability and adaptability are paramount.

Building on the mechanical insights provided by Nejad et al. (2024), the ISO 5840 standard serves as the definitive regulatory framework for ensuring that bioengineered heart valves meet essential durability and performance criteria (Stasiak et al., 2020). For surgically implanted valves, the standard requires a minimum of 200 million cycles under physiological conditions, simulating long-term use with systolic and diastolic pressures of 120 mmHg and 80 mmHg, respectively. Additional benchmarks include a mean pressure gradient below 10 mmHg and an effective orifice area (EOA) of at least 1.5 cm^2^ to guarantee adequate hemodynamic performance. In parallel, transcatheter heart valves (THVs) must endure 600 million cycles at pressures ≥140 mmHg, and demonstrate hemolytic indices <0.02% under high shear rates, ensuring long-term performance. With the valve’s visual design visible in Figure 8e, these stringent benchmarks establish critical targets that guide the design, testing, and eventual clinical translation of tissue-engineered heart valves.

Boehm et al. (2023) align closely with these standards, demonstrating a fiber-reinforced scaffold with high circumferential tensile strength (461.64 ± 58.87 N/m) and significant strain capacity (49.43% ± 7.53%), properties essential for meeting ISO-mandated durability and fatigue resistance. This data aligns with the patterns depicted in Figures 8a,b.

Materials such as silicone polyurethanes, polycarbonate urethanes (PCU), and nanocomposite polymers have played a central role in artificial heart valve development by addressing challenges of durability and biocompatibility. Silicone polyurethanes are known for their flexibility and chemical stability, while PCU combines strength and elasticity, providing a scaffold environment conducive to cell attachment and tissue integration. Nanocomposite polymers, enhanced with nanoparticles, improve mechanical properties and biocompatibility. However, Boehm et al. (2023) and Stasiak et al. (2020) demonstrate that further advancements like PVDF, SEPS, and SEBS within these material classes push the boundaries of performance while adhering to or exceeding ISO standards.

Boehm et al. (2023) showcase the mechanical robustness of bioengineered valves, meeting ISO 5840 standards with a circumferential failure stress of 461.64 ± 58.87 N/m and a strain of 49.43% ± 7.53%. The materials used not only ensures durability but also supports mechanical behavior that closely mimics native valve tissue. However, concerns persist regarding PVDF’s inherent stiffness and the potential for long-term degradation under physiological conditions, highlighting the need for extended *in vivo* evaluation and materials optimization.

Stasiak et al. (2020) demonstrate that polymeric heart valves (PHVs) exceed ISO 5840 standards with over 1.2 billion cycles in durability testing, equivalent to approximately 30 years of operation. These valves, constructed from SEPS and SEBS, achieve a balance between strength and flexibility, with polystyrene domains providing mechanical stability and elastomeric phases enabling dynamic motion.

These studies emphasize the promise of advanced materials such as PVDF, PCU, SEPS, and SEBS, while also highlighting the critical need for ongoing material innovation and rigorous testing to ensure sustained performance in the demanding cardiac environment.

#### 5.1.3 Hemocompatibility and biostability

Hemocompatibility is critical for the performance and longevity of heart valve scaffolds, as issues like thrombosis and calcification limit their clinical success. Subclinical leaflet thrombosis (SLT) prevalence ranges from 7% to 35% in bioprosthetic heart valves, while clinical valve thrombosis (CVT) rates are between 0.6% and 2.8% (Dangas et al., 2020). Liang et al. (2023) demonstrated that biomimetic modifications with CAG peptides - short chains of amino acids that promote endothelial cell adhesion and proliferation - and MPC, a phosphorylcholine-based polymer known for its anti-thrombogenic and anti-adsorption properties, can significantly improve scaffold hemocompatibility. Similarly, Liu et al. (2024) reported significant reductions in platelet adhesion and calcification using porcine pericardia modified with PDMAA, a hydrophilic polymer that inhibits protein adsorption and thrombus formation. These promising strategies could be explored for bioengineered heart valve scaffolds to address persistent challenges in hemocompatibility and durability. However, their application in tissue-engineered constructs requires further investigation to assess compatibility with living cells, scaffold biomechanics, and long-term clinical performance. Table 3 presents a comparison of key properties of scaffold materials and their main concerns for clinical applications.

The integration of advanced biomimetic materials into bioprosthetic heart valves offers a promising path to address longstanding issues such as thrombosis, calcification, and limited endothelialization. Approaches such as CAG (cysteine-alanine-glycine) peptides for improved endothelial cell adhesion, MPC (2-methacryloyloxyethyl phosphorylcholine) for enhanced anti-thrombogenic properties, and PDMAA (N, N-dimethylacrylamide) for reducing platelet adhesion and calcification have demonstrated significant potential (Liang et al., 2023) (Ding et al., 2022). However, their clinical application raises critical questions regarding long-term biocompatibility, mechanical stability, and scalability in tissue-engineered scaffolds. While these strategies effectively address key hemocompatibility challenges in controlled settings, their translation into fully functional, patient-specific bioengineered heart valves requires rigorous testing to balance biological integration with structural durability. Without these validations, their potential remains theoretical, underscoring the need for focused research to bridge the gap between innovation and clinical utility.

### 5.2 Tissue engineering and bioreactor systems

Building on the previous discussion of the biomaterials used to create scaffolds and the types of cells that can be seeded, this section focuses on the next critical steps: cell seeding and maturation. These processes are essential for determining how effectively cells adhere to the scaffold, proliferate, and differentiate into functional tissue. Traditional approaches typically involve passive cell seeding methods and static maturation conditions, but these techniques often struggle to achieve uniform cell distribution and maintain structural integrity. This section will explore how these methods work, their limitations, and their role in tissue development.

Cell seeding methods play a crucial role in tissue engineering, with passive and active techniques offering distinct outcomes. Passive seeding, where cells are deposited onto scaffolds without external assistance, often results in uneven cell distribution and compromised extracellular matrix (ECM) quality, limiting its effectiveness for applications such as cartilage tissue engineering (Giretova et al., (Giretová et al., 2019); Lv et al., (Lv et al., 2016)). In contrast, active seeding methods, such as centrifugation and static agitation, promote more uniform cell distribution, enhance extracellular matrix (ECM) formation, and improve mechanical properties. Research by Lv et al. (2016) demonstrates that these methods also facilitate better chondrogenic differentiation of mesenchymal stem cells (MSCs), positioning active seeding as the superior approach for applications in regenerative medicine.

Static seeding, a commonly employed passive method, involves pipetting a cell-media mixture directly onto scaffolds, followed by a 24-h incubation period to allow cell attachment (Saunders et al., 2022) (Saunders et al., 2022). While simple and accessible, static seeding often results in uneven cell distribution, as evidenced by scanning electron microscopy (SEM) analyses showing large cell patches on scaffold surfaces, with minimal infiltration into deeper regions (Saunders et al., 2022) (Blaudez et al., 2020). Histological evaluations further highlight cell aggregation and areas with insufficient attachment, which are critical drawbacks for tissue development (Saunders et al., 2022). Despite yielding relatively high metabolic activity in some conditions, static seeding fails to achieve uniform cellular distribution, particularly in low-porosity scaffolds, and limits internal cell density, impacting tissue integration and vascularization (Saunders et al., 2022; Blaudez et al., 2020). Consequently, while advantageous for its simplicity and use in high-porosity scaffolds, static seeding is less effective for applications requiring homogeneous cell spread and robust tissue formation.

Active cell seeding techniques are fundamental in tissue engineering, enabling precise cell distribution, enhanced adhesion, and scaffold functionality. Among these methods, perfusion seeding has demonstrated exceptional efficiency in optimizing cell placement through dynamic fluid flow, making it a preferred approach for complex tissue constructs. Zhang et al. (2020) emphasized its importance in porous scaffolds, utilizing a sophisticated numerical model to simulate cell mechanics, fluid interactions, and adhesion dynamics (Blaudez et al., 2020). This model revealed that factors such as perfusion pressure and initial cell concentrations significantly influence seeding efficiency, with higher perfusion velocities improving uniformity and adhesion rates. Engel et al. (2021) further supported this by developing a scalable perfusion bioreactor that achieved 100% endothelial cell seeding efficiency within vascular constructs after 13 days of smooth muscle culture, demonstrating not only uniform distribution but also sterility maintenance during sequential seeding (Engel et al., 2021). This efficiency was coupled with a more than three-fold increase in smooth muscle cell proliferation within 4 days and differentiated phenotypes by day 16, highlighting the bioreactor’s capacity to support growth and maturation (Engel et al., 2021). Additionally, collagen deposition reached 1.9% of dry weight, with glycosaminoglycan content at 19.2%, underscoring the bioreactor’s ability to promote extracellular matrix production critical for structural integrity (Engel et al., 2021).

In cardiac applications, Williams et al. utilized porous elastomeric scaffolds with 250 µm channels, mimicking natural capillary networks, to enhance perfusion and protect cells from shear stress (Williams and Wick, 2004). Their optimized perfusion velocity of 1.0 mm/s resulted in seeding efficiencies of 87% ± 26% for C2C12 myoblasts and 77.2% ± 23.7% for neonatal rat cardiac myocytes. Lower velocities, such as 0.1 mm/s, produced uneven distribution, reinforcing the importance of flow control for homogeneity (Williams and Wick, 2004). Sequential seeding methods, involving the initial placement of cardiac cells followed by endothelial cells, yielded a 76.1% ± 13.2% efficiency for endothelial attachment when using stacked scaffolds, ensuring open perfusion channels (Williams and Wick, 2004). Live/Dead assays further confirmed dense and viable cell populations post-seeding, indicating that dynamic perfusion conditions preserved cell health and functionality (Williams and Wick, 2004).

While static seeding presents a simpler and more economical alternative, it often results in uneven distribution and limited scaffold penetration, particularly in large or complex constructs. Its lower cost makes it suitable for smaller-scale applications, but it lacks the scalability and precision offered by perfusion-based systems (Williams and Wick, 2004). Perfusion seeding, by contrast, demonstrates superior outcomes across multiple studies, showing high cell viability, robust proliferation, and extracellular matrix deposition essential for tissue maturation and function.

Electrostatic seeding works through immobilizing primary cardiac myocytes within electrospun fibers, this technique enables the creation of patches that can repair, replace, and rejuvenate damaged heart tissue. These patches closely mimic the native architecture, supporting proper integration and function post-implantation. As this technology evolves, it holds significant promise for improving outcomes in regenerative medicine and treating heart disease (Ehler et al.) (Ehler and Jayasinghe, 2014).

Electrostatic seeding is one of the longest-standing techniques in cardiac tissue engineering, with studies on its potential dating back as far as 1996 (Brown et al., 1996). Despite its long history, challenges remain in optimizing electrospinning for cardiac patch development. The most significant hurdle lies in refining the electrostatic seeding process to ensure consistent and effective cell adhesion. Research by Mostafavi (2021) has shown that factors such as scaffold material, moisture levels, and electrical conductivity play crucial roles in enhancing the efficiency of electrospinning. The introduction of conductive additives has proven successful in aligning the conductivity of engineered patches with native myocardial tissue, making them more suitable for heart repair. However, more work is required to fine-tune the process, particularly in ensuring the survival and functionality of seeded cells within the scaffolds. The continuing development of electrostatic seeding will likely drive the future of cardiac tissue engineering, providing new solutions for heart regeneration and improving therapeutic outcomes for patients suffering from heart diseases (Basiry and Esehaghbeygi, 2012).

### 5.3 Additive manufacturing

Additive manufacturing (AM) has revolutionized cardiac tissue engineering by enabling the precise fabrication of complex structures for both *in vitro* models and regenerative therapies. Among its various techniques, stereolithography (SLA) stands out for its capacity to produce high-resolution, intricately patterned geometries, making it particularly valuable in cardiovascular applications. Esparza et al. (2023) demonstrated the utility of SLA by fabricating capillary-driven microfluidic devices, which provided controlled environments for studying cell alignment and tissue organization. While effective for *in vitro* modeling, their approach underscored the ongoing challenge of incorporating vascularization strategies to maintain tissue viability. Expanding on this work, Others (Lu et al., 2023) utilized 3D bioprinting to engineer vascularized cardiac tissues using human-induced pluripotent stem cell-derived cardiomyocytes (iPSC-CMs). Although promising, their constructs raised concerns about scalability and mechanical stability. More recently, Cui et al. (2023) developed SLA-based myocardial constructs featuring anisotropic myofiber alignment and perfusable vascular channels, significantly enhancing cardiac cell functionality. However, the use of novel bioink formulations in this approach introduces challenges related to reproducibility and clinical translation.

Together, these studies highlight the transformative potential of SLA and 3D bioprinting in cardiac tissue engineering, while emphasizing the importance of integrated approaches that balance structural precision, vascularization, and mechanical durability to drive clinical translation.

Another approach explored in the literature is Fused Deposition Modeling (FDM), which has gained traction in heart valve fabrication and broader tissue engineering applications. FDM enables the precise construction of scaffolds with customizable mechanical and biochemical properties, making it a valuable tool in the development of patient-specific implants (Cheung, 2018). As part of the broader field of bioengineering - which integrates engineering principles with biological sciences - FDM contributes to advancements in personalized medicine, regenerative therapies, and sustainable biomaterials. However, challenges such as high production costs, ethical considerations, and the complexity of biological systems can hinder widespread adoption. The chemical resistance and material variability of FDM-printed polyvinylidene fluoride (PVDF) structures, for example, are illustrated in Figure 9b (Rein et al., 2024), emphasizing the need for careful material selection and characterization when applying FDM in biomedical contexts.

Fused Deposition Modeling (FDM) is an additive manufacturing technique that builds complex 3D structures by depositing thermoplastic or biocompatible materials layer by layer. Its precision enables the fabrication of scaffolds with tailored geometries and mechanical properties, supporting cell growth and mimicking native tissue architecture. The method’s material versatility allows for the incorporation of bioactive components to enhance cell adhesion and integration. While FDM is scalable for larger regenerative applications, its high processing temperatures can compromise cell viability, posing a significant limitation (Kumar et al., 2024). Coulter et al. demonstrated FDM’s potential in creating heart valves with controlled stress distribution and mechanical integrity. However, optimizing leaflet thickness to improve hemodynamic performance remains a significant challenge (Coulter et al., 2019). Schroter et al. advanced these findings with the TIPI valve, leveraging 3D printing to achieve better hemodynamic metrics and address thrombogenic risks through innovative design modifications. The valve created and tested under pressure can be seen in Figure 9a. These studies emphasize FDM’s transformative role in tissue engineering while highlighting the ongoing challenges of balancing structural and biological functionality in engineered constructs (Schröter et al., 2023).

Comparing stereolithography (SLA) and fused deposition modeling (FDM) is essential for understanding their respective strengths and limitations in cardiac tissue engineering. Table 4 illustrates the differences between stereolithography and fused deposition modeling (FDM). SLA offers high precision and the ability to fabricate intricate geometries, making it ideal for advanced *in vitro* models. In contrast, FDM provides greater scalability and material versatility, which are critical for producing larger constructs such as heart valves. Evaluating these techniques side by side enables researchers to make informed choices that optimize structural integrity, biological functionality, and clinical applicability, ultimately advancing the field toward real-world solutions.

**TABLE 4:** Stereolithography compared with fused deposition modeling.

Aspect	Stereolithography (SLA)	Fused deposition modeling (FDM)
Technique	Uses light to cure resin layer by layer, allowing for high-resolution geometries (Esparza et al., 2023; Cui et al., 2023)	Deposits thermoplastic or biocompatible material layer by layer to create 3D structures (Cheung, 2018; Kumar et al., 2024)
Applications	Primarily used for fabricating microfluidic devices, vascularized cardiac tissues, and anisotropic myofibers (Esparza et al., 2023; Lu et al., 2023; Cui et al., 2023)	Widely employed in heart valve fabrication and scaffold design for tissue engineering (Coulter et al., 2019; Schröter et al., 2023; Kumar et al., 2024)
Resolution	High precision enables intricate geometries suitable for *in vitro* modeling and regenerative therapies (Esparza et al., 2023; Cui et al., 2023)	Moderate precision, with a focus on tailored mechanical and biochemical properties (Cheung, 2018; Kumar et al., 2024)
Material Versatility	Relies on bioinks that enhance cellular functionality, but novel formulations pose challenges for reproducibility (Cui et al., 2023)	Supports a variety of thermoplastics and biocompatible materials, allowing for bioactive component integration (Cheung, 2018; Kumar et al., 2024)
Scalability	Limited scalability due to complex fabrication and material constraints (Cui et al., 2023)	High scalability supports larger constructs for regenerative applications (Cheung, 2018; Kumar et al., 2023)
Challenges	Incorporating vascularization strategies for tissue viability remains a key challenge (Esparza et al., 2023)	High temperatures required during fabrication can reduce cell viability (Kumar et al., 2024)
Innovations	Recent developments include perfusable vascular channels and anisotropic constructs (Cui et al., 2023)	Advances include the TIPI valve with improved hemodynamic performance and reduced thrombogenic risks (Schröter et al., 2023)
Clinical Translation	Focuses on balancing structural precision with vascularization for functional cardiac tissues (Lu et al., 2023; Cui et al., 2023)	Aims to optimize structural and biological functionality in constructs such as heart valves (Coulter et al., 2019), (Schröter et al., 2023)

## 6 Advancements, future directions, and conclusive remarks

**FIGURE 10:**
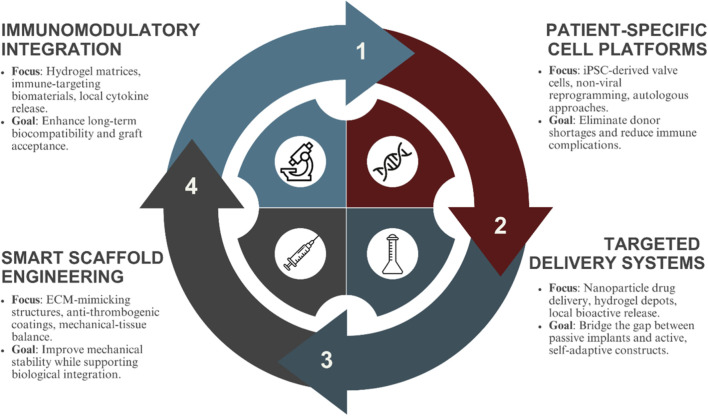
Future directions in bioengineered heart valves. Integration of immune engineering, scaffold design, and patient-specific approaches to advance personalized TEHV therapy.

Tissue-engineered heart valves (TEHVs) offer a promising alternative to mechanical and bioprosthetic valves by addressing critical limitations such as thrombogenicity, structural degeneration, and lack of growth potential. While substantial progress has been achieved - through immunomodulation (Latis et al., 2019), biocompatible platforms (Abbaszadeh et al., 2023; Nadine et al., 2021) and careful use of biostable polymers (Dangas et al., 2020; Liang et al., 2023) - several challenges continue to impede clinical translation. Lídia et al. (2021) demonstrated exceptional preservation of the extracellular matrix (ECM), enhancing durability and promoting cellular integration, while Boehm et al. (2023) set new performance benchmarks in accordance with ISO 5840 standards. Despite these advances, issues such as immune rejection and limited long-term functionality remain major obstacles. Future directions and the key steps required in this field are illustrated in Figure 10.

Recent studies highlight critical areas for future focus. Liang et al. (2023) emphasized the urgent need to improve hemocompatibility and biostability to reduce risks of thrombosis and calcification, which are among the most pressing barriers to successful TEHV implantation. Singh et al. (2013) further argued that careful optimization of cell sources and biomaterial compositions is essential for modulating immune responses and promoting seamless graft integration. In summary, a multidisciplinary approach, combining advanced material science, immune engineering, and *in vivo* validation, is likely to be essential for overcoming current limitations. However, empirical validation and large scale *in vivo* validation is still largely required in the field.

One significant barrier in regenerative medicine remains the ethical controversy surrounding embryonic stem cells (ESCs) (Volarevic et al., 2018). In contrast, induced pluripotent stem cells (iPSCs) offer a promising alternative, free from ethical concerns and with reduced risk of immune rejection (Amabile and Meissner, 2009). Although iPSC generation typically involves the reprogramming of adult cells using viral vectors (Albert and Butcher, 2023), they represent a scalable and clinically relevant solution to the persistent shortage of donor valves.

Beyond cell sourcing, scaffold design must also continue to evolve. The delivery of cytokines *via* nanoparticles and hydrogels has shown significant potential in promoting heart tissue regeneration and enhancing integration with native tissue (Cheung, 2018). Furthermore, immunomodulatory platforms, ranging from cell-based therapies to hydrogel matrices, have demonstrated the ability to regulate immune responses effectively (Coulter et al., 2019; Ruth, 2024). These strategies not only enhance biocompatibility, but also help minimize long-term complications, making bioengineered heart valves a more viable and adaptive therapeutic option.

Looking forward, several advancements in the field have already emerged. In biomaterials, following the emergence of hydrogels, several hydrogels are being developed to increase the biocompatibility of TEHVs (Chen et al., 2025). Furthermore, natural polymers such as polysaccharides and proteins are being used in scaffolds to mimic native heart tissue properties (Ciolacu et al., 2022). With immunomodulation, biodegradable polymers and hydrogels have shown promise in reducing immune response, reducing needs for immunosuppressants altogether (Esmaeili et al., 2025). Although this has yet to be clinically tested, as gene edited technologies such as CRISPR emerge, we can also look to utilize them for use of iPSC, ESC, and MSCs to reduce their tumorigenic potential and enhance regenerative qualities.

For tissue-engineered valves to move beyond experimental success, these immunomodulatory techniques must undergo standardized, large-scale preclinical evaluation and human trials. By addressing immune compatibility at every stage - from cell selection to scaffold integration - bioengineered valves can move beyond passive replacements and become active, adaptive constructs that function seamlessly within the host environment.
